# Additive Toxicity of β-Amyloid by a Novel Bioactive Peptide *In Vitro*: Possible Implications for Alzheimer’s Disease

**DOI:** 10.1371/journal.pone.0054864

**Published:** 2013-02-04

**Authors:** Sara Garcia-Ratés, Matthew Lewis, Rosemary Worrall, Susan Greenfield

**Affiliations:** Department of Pharmacology, University of Oxford, Oxford, United Kingdom; Biological Research Centre of the Hungarian Academy of Sciences, Hungary

## Abstract

**Background:**

β-amyloid is regarded as a significant factor in Alzheimer’s disease: but inefficient therapies based on this rationale suggests that additional signalling molecules or intermediary mechanisms must be involved in the actual initiation of the characteristic degeneration of neurons. One clue could be that acetylcholinesterase, also present in amyloid plaques, is aberrant in peripheral tissues such as blood and adrenal medulla that can be implicated in Alzheimer’s disease. The aim of this study was to assess the bioactivity of a fragment of acetylcholinesterase responsible for its non-enzymatic functions, a thirty amino acid peptide (“T30”) which has homologies with β-amyloid.

**Methods:**

Cell viability was measured by sulforhodamine B assay and also lactate dehydrogenase assay: meanwhile, changes in the status of living cells was monitored by measuring release of acetylcholinesterase in cell perfusates using the Ellman reagent.

**Findings:**

T30 peptide and β-amyloid each have toxic effects on PC12 cells, comparable to hydrogen peroxide_._ However only the two peptides selectively then evoke a subsequent, enhanced release in acetylcholinesterase that could only be derived from the extant cells. Moreover, unlike hydrogen peroxide, the T30 peptide selectively shifted a sub-threshold dose of β-amyloid to a toxic effect, which also resulted in a comparable enhanced release of acetylcholinesterase.

**Interpretation:**

This is the first study comparing directly the bioactivity of β-amyloid with a peptide derived from acetylcholinesterase: the similarity in action suggests that the sequence homology between the two compounds might have a functional and/or pathological relevance. The subsequent enhanced release of acetylcholinesterase from the extant cells could reflect a primary ‘compensatory’ response of cells prone to degeneration, paradoxically providing further availability of the toxic C-terminal peptide to modulate the potency of β-amyloid. Such a cycle of events may provide new insights into the mechanism of continuing selective cell loss in Alzheimer’s disease and related degenerative disorders.

## Introduction

Although it is well accepted that β-Amyloid (Aβ) plays an important role in the pathogenesis of Alzheimer’s disease (AD), its precise contribution to the basic mechanism of neurodegeneration remains questionable. For example, although the brains of patients with AD contain deposits of Aβ, so too can those from healthy controls [1]. Hence if Aβ is a necessary but not sufficient factor, could there be an endogenous agent that determines the eventual toxicity?

Acetylcholinesterase (AChE) is contained in all the vulnerable central nervous system (CNS) neurons prone to neurodegeneration, irrespective of the transmitter they use: we have therefore proposed that the neurotoxic actions of the C-terminal peptides derived from AChE as a signal molecule could be the common mechanism for Parkinson’s disease, Motor neurone disease and AD [2].

**Figure 1:**
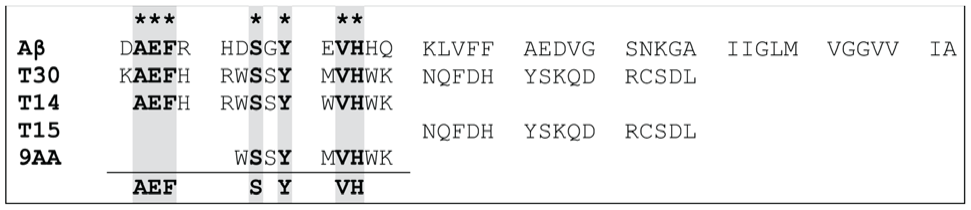
Alignment of the sequences of β-Amyloid (Aβ) compared with the AChE C-terminus peptides of 30 amino acids (T30), 14 amino acids (T14), and the residual 15 amino acid sequence between the two (T15). Note homology between the 9 amino acid sequence (9AA) possible in human plasma and T14, T30 and Aβ (shaded panels), but not T15.

Within the C-terminal amino acid sequence of the AChE the 14 mer T14 is the active region that has a close homology to the sequence of Aβ (Fig. 1). It is noteworthy that a nine amino acid sequence within the 14 mer sequence has been listed as having a high probability of occurring in biological tissues, specifically human plasma [3]. However the longer 30 amino acid molecule, that includes T14, has proved the more potent in its activity due to its greater stability [4], and hence was the molecule of choice in this study. The residual 15 amino acid sequence, T15, was used as a control for non-specific peptide effect [2]. These peptides (T14 and T30) mimic the ‘non-cholinergic’ actions reported previously, have effects that can be both acute and chronic, and range along a trophic-toxic axis of excitotoxicity depending on duration and dose of administration, in a wide range of preparations [5].

One particularly appropriate preparation for studying possible mechanisms of toxicity, are chromaffin cells. Since chromaffin cells are derived from the neural crest but are located in the centre of an accessible peripheral organ (the adrenal medulla) they have been described as offering a ‘window’ into the brain [6]. These cells could serve as a powerful, albeit novel, *in vitro* model for studying the still unknown primary process of neurodegeneration: the adrenal medulla in AD patients shows various pathological features reminiscent of those seen in the CNS e.g. numerous Lewy-body like inclusions [7], neurofibrillary tangles and paired helical filaments [8], as well as expression of amyloid precursor protein (APP) [9]. Interestingly enough Appleyard and Macdonald [10] have demonstrated a selective reduction only in the soluble i.e. releasable form of AChE from the adrenal gland in AD, perhaps due to its enhanced secretion into the plasma, where it is elevated in AD patients [11], [12]. Accordingly, it may be the case that AChE has an alternative, non-enzymatic function [13], [14], [15], as a signalling molecule in its own right [16].

PC12 cells are a cloned, pheochromocytoma cell line derived from the adrenal medulla [17], [18]. They are easily cultured and readily accessible to experimental manipulations. Moreover, given that cell lines derived from the chromaffin cells of the adrenal medulla are indeed a powerful *in vitro* tool, the aim of this study was to see whether the effects of bioactive agents, ie C-terminal peptides and also Aβ, can be reflected in changes in cell viability and subsequent release of AChE.

## Materials and Methods

### PC12 Cell Culture

Wild-type PC12 cell were provided by Sigma-Aldrich (St. Louis, MO). The culture was routinely plated in 100 mm dishes (Corning) coated with collagen and maintained in growth medium with Minimum Essential Medium Eagle (MEM) supplemented with heat-inactivated 10% horse serum and 5% foetal bovine serum, 10 mM HEPES, 2 mM glutamine and 0.25 ml Penicillin/streptomycin solution. Cells were maintained at 37°C in a humidified atmosphere 5% CO_2_ and the medium was replaced every 2 days. For splitting, cells were dislodged from the dish using a pipette with medium, with a portion of these replated onto new cultured dishes. Cells were used between passages 12 and 19.

### Treatment of Cells

In the first instance, in order to assess the toxicity of the C-terminal peptide on cell viability, cells were treated with increasing concentrations of T30 (from 0.2 µM to 2 µM) for 1 and 6 hours. Controls were administered distilled water. The desired amount of drug was taken from stock solutions diluted in distilled water and added to each well containing 200 µl of growth medium. For the following cell viability experiments, the T30 concentration of 0.7 µM was chosen because it was the lowest concentration with the maximum effect. In order to compare the effects of T30 with those of Aβ (1–42) for 15 minutes and 1 hour, an experiment with the same concentration of Aβ (1–42) (0.7 µM) was performed. As a positive control of cell death hydrogen peroxide (H_2_O_2_) was used at a concentration of 100 µM and T15 0.7 µM was used as a negative control. On the other hand, to study the additive effect of the two molecules, an experiment with subthreshold concentrations of Aβ (1–42) was performed with the following treatments: Ctrl, T30 0.1 µM, T30 0.7 µM, Aβ (1–42) 0.01 µM, Aβ (1–42) 0.1 µM, T30 0.7 µM+Aβ (1–42) 0.01 µM and T30 0.7 µM+Aβ (1–42) 0.1 µM.

For the LDH experiments, cells were pretreated as for the cell viability experiments: Ctrl, T30 0.7 µM, Aβ (1–42) 0.7 µM and H_2_O_2_ 100 µM for 1 hour.

For AChE activity, in one experiment cells were treated as for the viability study: Ctrl, T15 0.7 µM, T30 0.7, Aβ (1–42) 0.7 µM and H_2_O_2_ 100 µM for 15 minutes and 1 hour. In the second experiment, in order to study the additive effects of T30 and Aβ (1–42) on AChE activity, cells were treated as follows: Ctrl, T30 0.1 µM, T30 0.7 µM, Aβ (1–42) 0.01 µM, Aβ (1–42) 0.1 µM, T30 0.7 µM+Aβ (1–42) 0.01 µM and T30 0.7 µM+Aβ (1–42) 0.1 µM. For these experiments, the desired amount of drug was taken from stock solutions diluted in distilled water and added to each well containing 200 µl of MEM and L-Glutamine. Serums were not added because they interfere in the detection of the AChE activity but do not affect cell viability.

### Morphological Observation

Morphological changes in the cells treated with T30 0.7 µM were determined by microscopic examination. Cells were visualized (40 X) using a Leitz Diaplan microscope and images were captured with a Leica DFC300FX digital camera and Leica Twain imaging software (Leica Microsystems Ltd., Milton Keynes, UK).

### Cell Viability Assay

The cell viability assay used was the sulforhodamine B (SRB) colorimetric assay for toxicity screening [19]. The day before of the experiment cells were seeded onto collagen-coated 96-well plates (Corning) in a concentration of 40.000 cells/well. Cell concentration was determined by the Fuchs-Rosenthal chamber. After treatment, medium was replaced and cells were fixed by adding 100 µl of 10% Trichloroacetic Acid (TCA) for 1 h at 4°C. Thereafter, cells were washed with H_2_O and stained with 100 µl of a 0.057% SRB solution in 1% Acetic acid (HAc) for 30 minutes at room temperature. After staining cells were washed with 1% HAc for removing the excess of SRB and then incubated with 200 µl of 10 mM Tris base, pH 10.5 and shake it for 5 minutes to solubilise the protein-bound dye. Measurement of the absorbance took place in a V_Max_ Kinetic Microplate Reader (Molecular Devices) at 490 nm.

### Lactate Dehydrogenase as a Measure of Cellular Citotoxicity

Lactate dehydrogenase (LDH) is a cytoplasmic enzyme that catalyses the conversion of lactate to pyruvate. As LDH is released from cells following a toxic stimulus that causes membrane dissolution, the quantification of LDH activity in perfusates was used to measure cell lysis [20]. During the reduction of pyruvate an equimolar amount of reduced nicotinamide adenine dinucleotide (NADH) is oxidised to nicotinamide adenine dinucleotide (NAD^+^), resulting in a decrease of light absorbance at 340 nm. After treatment, supernatant (perfusate) of each treatment was collected and 40 µL of each condition were added to a new flat bottomed 96 well plate followed by the addition of 80 µl of pyruvate solution (16.2 mM) and 80 µl of NADH (0.191 mM). Measurement of the absorbance took place after 1 hour in a Fluostar Optima Microplate Reader (BMG) at 340 nm.

### Acetylcholinesterase Activity Assay

AChE activity was measured using the Ellman reagent that measures the presence of thiol groups as a result of AChE activity [21]. Cells were plated the day before the experiment as for the cell viability assay. After treatment, supernatant (perfusate) of each treatment was collected and 25 µL of each condition were added to a new flat bottomed 96 well plate followed by the addition of 175 µl of Ellman reagent (Solution A: KH_2_PO_4_ 139 mM and K_2_HPO_4_ 79.66 mM, pH 7.0; solution B (substrate): Acetylthiocholine Iodide 11.5 mM; Solution C (Reagent): 5, 5′-Dithiobis (2-nitrobenzoic acid) 8 mM and NaHCO_3_ 15 mM). The Ellman reagent was prepared as a mixture of the 3 solutions in a ratio 33(A):3(B):4(C). Absorbance measurements were taken at regular intervals (3, 10, 30 and 60 mins) across experiments at 405 nm.

### Drugs and Reagents

MEM, culture serums, antibiotics, collagen, sulforhodamine B, Aβ (1–42), NADH, piruvate and buffers reagents were provided by Sigma-Aldrich (St. Louis, MO). T30 and T15 AChE peptides were synthesized by Genosphere Biotechnologies (Paris, France). Stocks of peptides were diluted in distilled water.

### Data Analysis

For each experiment, the mean of the absorbance of each condition was calculated followed but the calculus of the percentage of each value related to its own control. The statistics analysis was performed with the average of the percentage values of three or more experiments. Comparisons between multiple treatment groups and the same control were performed by one-way analysis of variance (ANOVA) and Tukey’s post-hoc tests using GraphPAD Instat (GraphPAD software, San Diego, CA). These tests compare the means of every treatment to the means of every other treatment; that is, apply simultaneously to the set of all pairwise comparisons and identify where the difference between two means is greater than the standard error would be expected to allow. Statistical significance was taken at a *P* value <0.05. Graphs were plotted using GraphPAD Prism (GraphPAD software, San Diego, CA). In the case of the dose response experiment, the EC_50_ values were calculated by fitting the logarithm of the experimental data points to a single site Hill equation using a non-linear regression curve using GraphPad Prism.

## Results

### Dose-response Effect of T30 on PC12 Cell Viability

**Figure 2:**
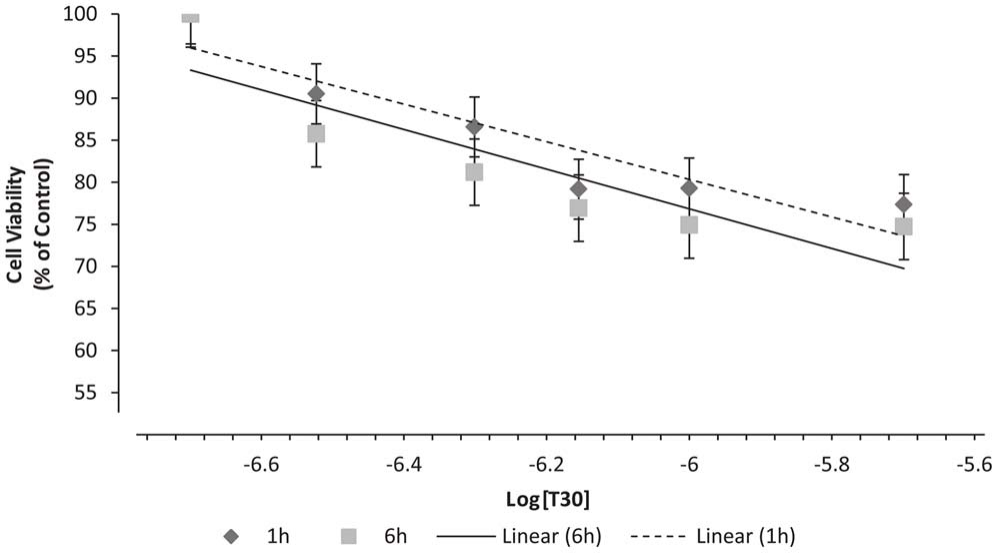
Dose-response curves representing cell viability (% of control) after 1 and 6 h of treatment with AChE C-terminus peptides of 30 amino acids (T30) with concentrations. Data are presented as the percentage (of control) of the mean ± S.E.M. from five independent experiments carried out on triplicates (*P<0.05, N = 5).

**Figure 3:**
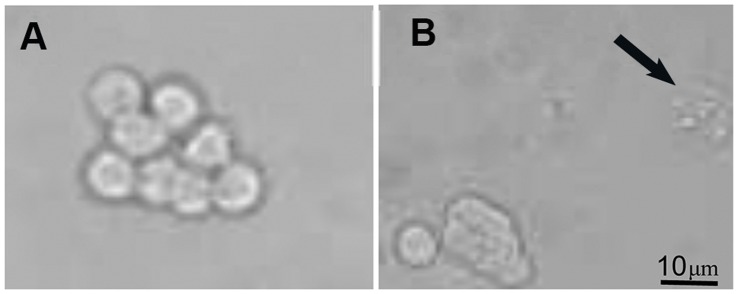
Effect of AChE C-terminus peptides of 30 amino acids (T30) on morphology of PC12 cells. (A) Control cells presented a round shape with a big nucleus. In contrast, (B) Cells treated with T30 presented granulation of the nucleus () as apoptotic feature.

Undifferentiated PC12 cells were treated with T30 for 1 and 6 hours to assess the effect on cell viability. In both cases, T30 had a dose-dependent effect reducing the viability of the cells (Fig. 2): *0,2 µM:* 100% ±7.8% & 100% ±5.11%, *0.3 µM:* 90.505% ±5.415% & 85.77% ±4.1%, *0.5 µM:* 86.575% ±4.62% & 81.193% ±5.76%, *0.7 µM*: 79.175% ±3.36% & 76.92% ±3.9%, *1 µM:* 79.305% ±5.31% & 74.92% ±3.9%, *2 µM:* 77.36% ±9.22% & 74.743% ±3.5%; 1 & 6 h respectively. There were no significant difference between 1 and 6 hours of treatment, and the effect of T30 on cell viability seemed to plateau when compared with H_2_O_2_ (*1h*: 74.49% ±4.17%, *P<*0.01 and *6* *h:* 42.67% ±0.83%, *P*<0.001). The highest concentration of T30 used, 2 µM, reduced cell viability by about 25% in both cases (*1* *h*: 77.36% ±9.22%, *6* *h*: 74.743% ±3.5%). The EC_50_ values obtained from 1 and 6 h were 0.8604±0.08 µM and 0.573±0.107 µM, respectively. T15 (2µM) had no effect on cell viability (*1* *h:* 93.44% ±8.19% and *6* *h*: 96.06% ±8.3%). Morphological differences between control and treated cells can be observed in figure 3. Cells treated with T30 0.7 µM for 1 hour presented apoptotic features.

### Effect of T30 on PC12 Lactate Dehydrogenase Activity

**Figure 4:**
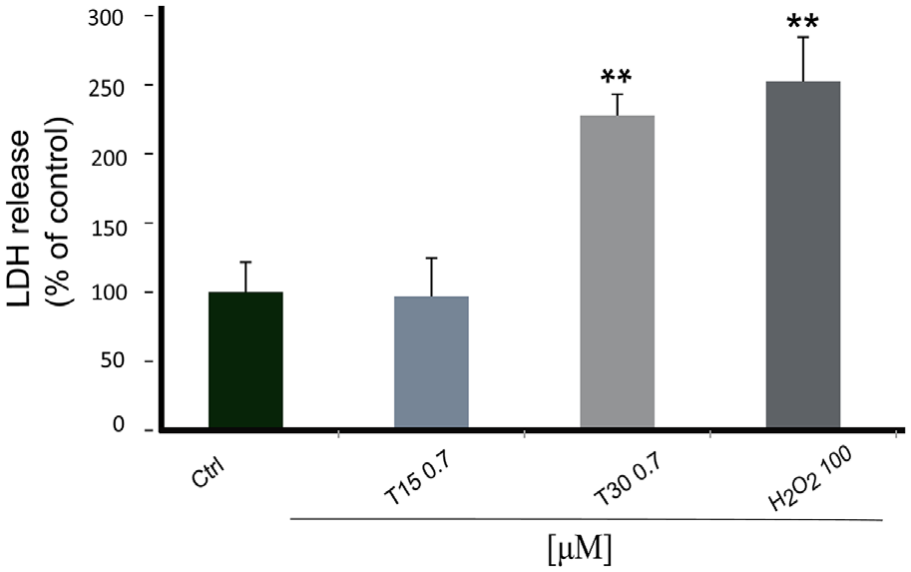
Bar graph showing lactate dehydrogenase release. Cells were treated for 1 hour with AChE C-terminus peptides of 30 amino acids (T30) 0.7 µM, the residual 15 amino acid sequence (T15) 0.7 µM and hydrogen peroxide (H_2_O_2_) 100 µM. Data are presented as the percentage (of control) ± S.E.M. (N = 3). * vs Control: **P<0.01.

In order to compare methodologies, the lactate dehydrogenase (LDH) assay was performed to monitor membrane integrity as a measure of cell viability. Both T30 and H_2_O_2_ induced a highly significant increase in LDH release (*T30:* 227.69% ±15.38, *H_2_O_2_:* 252.3% ±32.1; *P<*0.01). By contrast, T15 had no effect on LDH release (96.92% ±27.69%) (Fig. 4).

### Comparison between AChE Peptides and Aβ on Cell Viability

There were no significant differences between either control and T15 (*15 min*: 96.06% ±8.3%, *60 min*: 92.828% ±4.23%) or between control and 15 minute treatments with T30 and Aβ (*T30:* 83.61% ±6.02, *Aβ:* 87.65% ±6.2%). Surprisingly, after 60 minutes, Aβ reduced cell viability by about 25% with a similar effect to T30 (*T30*: 76.92% ±3.9, *Aβ*: 75.03% ±3.81%; *P*<0.01) (Table 1).

**Table 1:** Representative table of cell viability (% of Control) after 15 and 60 minutes treatments with Hydrogen peroxide (H_2_O_2_) 100 µM, β-Amyloid (Aβ) 0.7 µM, AChE C-terminus peptides of 30 amino acids (T30) 0.7 αM and the residual 15 amino acid sequence (T15) 0.7 αM. Data are presented as the percentage of the mean ± S.E.M. from five independent experiments carried out on triplicates (N = 5).

	H_2_O_2_	Aβ	T30	T15
**15 min**	81.075±6.675*	87.65±6.2	83.61±6.02	96.06±8.3
**60 min**	73.925±3.63**	75.03±3.81**	76.92±3.9**	92.828±4.23

* *P*<0.05 and **P<0.01 vs Control.

### T30 and Aβ Additive Effect on Cell Viability

**Figure 5:**
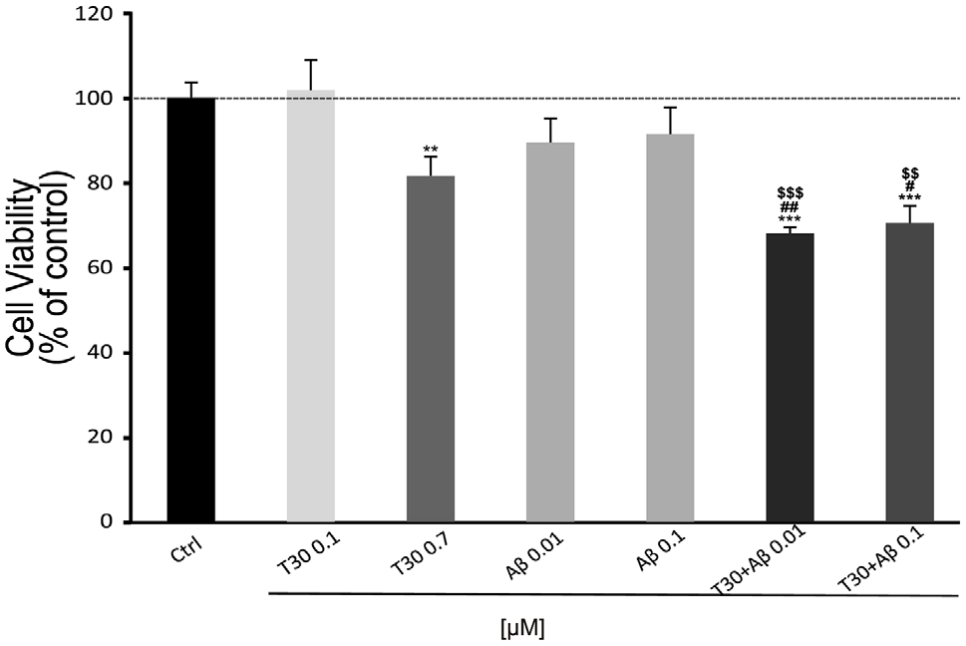
Bar graph showing the additive effect of β-Amyloid (Aβ) and AChE C-terminus peptides of 30 amino acids (T30) on cell viability. Cells were treated for 1 hour. Data is presented as % cell viability ± SEM (N = 3). * vs Control: **P<0.01, **P<0.001; ^#^ vs T30: ^##^ *P*<0.01, ^###^ *P*<0.001;^ &^ vs Aβ: ^&^ *P*<0.05, ^&&^ *P*<0.01.

After 1 hour, T30 0.7 µM reduced cell viability (81.54% ±4.5%, *P*<0.01) but a concentration of 0.1 µM of T30 and, 0.01 and 0.1 µM of Aβ were used and had no effect on cell death (*T30 0.1 µM*: 101.76% ±7.04%, *Aβ 0.01 µM*: 89.43% ±5.63%; *0.1 µM*: 91.4% ±6.3%). By contrast, when these concentrations of Aβ were applied together with T30 at 0.7 µM, the decrease in cell viability was potentiated by about 30% showing the additive effect of these two compounds (*T30+ Aβ (0.7/0.01 µM*: 67.95% ±1.47%, *0.7/0.1:* 70.42% ±4.08%; *P<*0.001). T15 was used as a control (*T15 0.7 µM*: 108.59% ±3.8%) (Fig. 5).

### T30 and Aβ Increase AChE Activity

**Figure 6:**
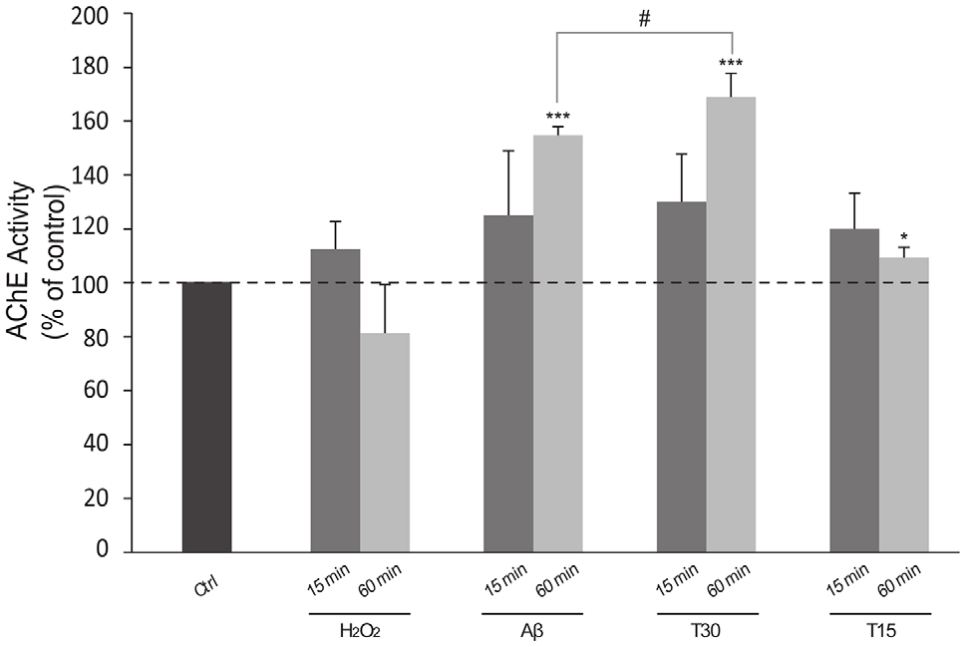
Bar graph shows the increase of Acetylcholinesterase (AChE) activity. Cells were treated for 15 and 60 minutes with Hydrogen peroxide (H_2_O_2_) 100 µM, β-Amyloid (Aβ) 0.7 µM, AChE C-terminus peptides of 30 amino acids (T30) 0.7 µM and the residual 15 amino acid sequence (T15) 0.7 µM. Data are presented as the percentage of the mean ± S.E.M. divided by the number of extant cells and obtained from independent experiments carried out on triplicates. *P<0.05 and ***P<0.001 vs Control; # *P*<0.05 between T30 treatments. Dotted line shows basal AChE activity (Control 100%).

There were no significant differences between 15 minutes treatments (*T15:* 120% ±13.3, *Aβ:* 125.07% ±24.027%, *T30:* 130% ±17.79%). However, data from the 60 minute treatment showed a significant increase in AChE activity after treatment with T30 and Aβ (Fig. 6) (*T15:* 109.35% ±3.804%, *Aβ:* 154.69% ±3.213%, *T30:* 168.83% ±8.85%; *P*<0.001). In order to demonstrate that the low levels of AChE activity after H_2_O_2_ treatment (*15 min:* 112.48% ±10.226%; *60 min:* 81.28% ±17.943%) were not due to AChE degradation, controls with different forms of AChE and H_2_O_2_ were performed showing that the AChE activity levels were no different from AChE alone (data not shown).

### T30 and Aβ Additive Effect on AChE Release

**Figure 7:**
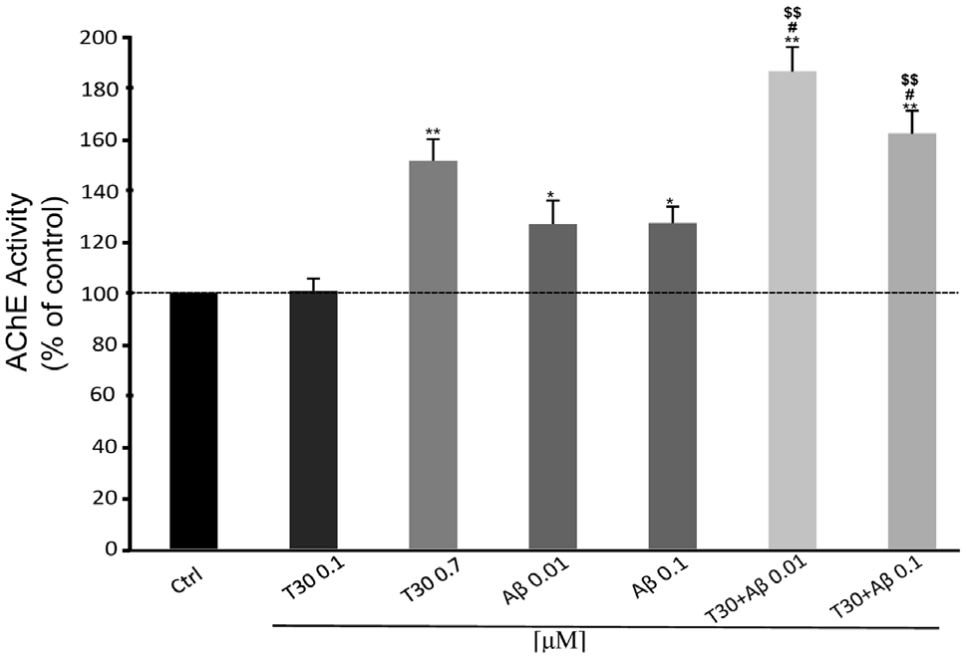
Bar graph showing the additive effect of β-Amyloid (Aβ) and AChE C-terminus peptides of 30 amino acids (T30) on Acetylcholinesterase (AChE) activity. Cells were treated for 1 hour. Data are presented as the percentage of the mean ± S.E.M. divided by the number of extant cells and obtained from independent experiments carried out on triplicates. *P<0.05 and **P<0.01 vs Control; ^#^ *P*<0.05 vs T30 0.1; ^$$^ *P*<0.01 vs Aβ. Dotted line shows basal AChE activity (Control 100%).

As previously seen in the cell viability assay, subthreshold concentrations of T30 and Aβ applied together induce an additive effect of increasing the activity of AChE by about 20% in cells perfusates after 1 hour treatment (Fig. 7) (*T30 0.1 µM:* 100.67% ±4.83%, *T30 0.7 µM:* 168.83% ±8.85% *P*<0.01, *Aβ 0.01 µM:* 126.83% ±9.17% *P*<0.05, *Aβ 0.1 µM:* 127.2% ±6.48% *P*<0.05, *T30 0.1+ Aβ 0.01:* 186.47% ±9.5% *P*<0.01, *T30 0.1+ Aβ 0.1:* 162.24% ±8.83% *P*<0.01).

## Discussion

The AChE terminal fragment T30, but not the control peptideT15, had a dose-dependent effect on cell viability from doses in the nanomolar range onwards. Previous studies have all shown bioactivity of both T14 and T30 peptides in the nanomolar range [22]–[25] and hence 700 nM was the dose subsequently used here as having maximal effect at the most physiological concentration. This effect, seen in the SRB assay, was confirmed by an alternative evaluation ie assay of perfusate for the soluble cytoplasmic enzyme, LDH. Application of T30 had a maximum effect of 25% cell loss, after one hour, ie had no further effect for the remaining period measured (6 hours): this observation of a plateau effect suggests that the peptide was having its action via a specific, and therefore limited target [26], [27] on the cell surface rather than as a result of non-specific interaction with the cell membrane generally. Aβ and T30 performed similarly after one hour, at comparable concentrations of 700 nM. Moreover the observation that the two different bioactive agents were additive in toxicity when applied together in erstwhile subthreshold doses, suggests a functional inter-relationship.

A physiological relevance of this effect is suggested by the subsequent observation of an enhancement in release of AChE by T30. Acetylcholinesterase release as a result of an apoptotic stimulus on PC12 cells has been previously reported [28]. As might be expected from a cell line derived from the adrenal medulla, where AChE secretion has already been observed, there was a stable baseline release of AChE from PC12 cells: hence we can conclude that the cells were in good health. Furthermore, T30, but not T15, had an action after one hour, whilst H_2_O_2,_ was ineffective: this observation therefore suggests that the selective action of T30 was not the result of leakage from the dead cells, but a physiological phenomenon where the enhanced release must have occurred from the 70% extant cells. The latency of one hour observed in this effect supports the scenario of a physiological ‘compensation’ on behalf of the cells still living. In addition, we report here for the first time a similarity in the bioactivity of T30 and Aβ, in enhancing release of AChE. A possible association has already been demonstrated in various studies between AChE and Aβ [29]. Also, it has been reported [30] that background of increased AChE levels could act as a nucleating factor promoting the conversion of Aβ from soluble to insoluble fibrils which induces plaque formation [31]. However this is the first report of an additive effect with amyloid and the peptide fragment of AChE believed to underlie its non-traditional, non-cholinergic actions.

Previous data from our group [32] have established that T30 interacts with the α7nAChR, a target also for Aβ [33]: hence a likely explanation of the additive effects seen on cell viability could well be at the level of this receptor [34].

**Figure 8:**
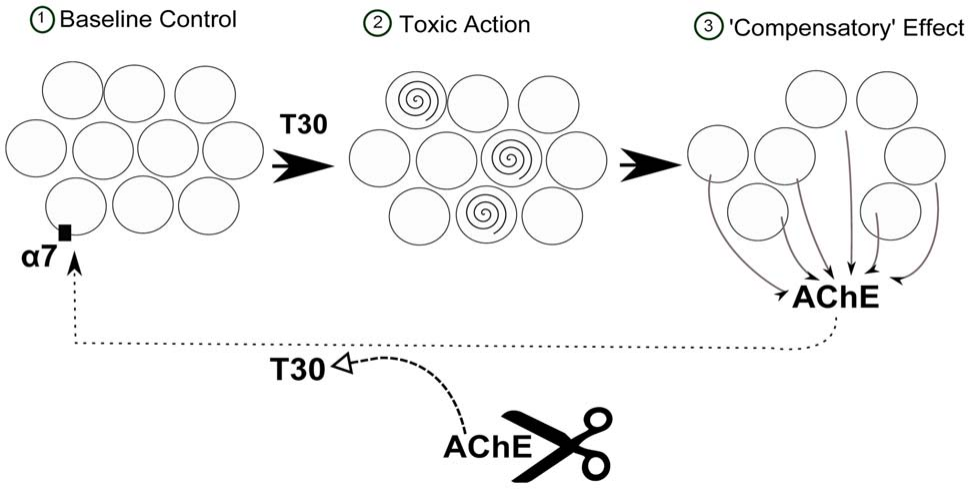
Possible scheme of the effects of T30 on PC12 cells. The population of cells (1) is treated with AChE C-terminus peptide of 30 amino acids (T30), causing a reduction of 30% of the population (2) and a consequent increase in AChE release from the 70% of the extant population as a ‘compensatory’ effect (3). The released AChE could be cleaved to yield T30 which then would act at the α7-nAChR, completing a feed-forward cycle of toxicity.

In conclusion, the data presented here may provide some further insight into the sequence of events in neurodegeneration: a possible scheme is shown in Figure 8. ‘Compensation’ within key neuronal populations has already been considered in the early stages of neurodegeneration [35]. Similarly, the lengthy delay between the onset of cell loss in AD and eventual appearance of symptoms [36], may also be indicative of compensatory mechanisms at work. We have previously proposed that it is these very mechanisms of ‘compensation’ that could constitute the remorseless cycle of cell death in neurodegenerative disorders [4].

Perhaps not surprisingly various questions then arise. First, why should the C-terminal peptide be the culprit rather than the whole AChE protein? AChE has indeed for many years been implicated and pharmaceutically targeted in AD and more recently in the formation of amyloid, by virtue of its enzymatic action: in contrast the C-terminal peptide would appear to operate in a non-cholinergic role as an independent entity. It has been reported that a form of AChE (a monomer lacking the peptide) is disproportionately dominant over the usual tetramer (containing the peptide) in development and that this situation is recapitulated in AD [37]: the most obvious explanation for this finding is that in both development and again in AD, the C-terminal peptide has been cleaved to operate as a signalling molecule in its own right. Moreover, the integrity of the C-terminal is required for the formation of disulphide bonds between the catalytic subunits of AChE, and this accounts for the fact that when they are present AChE always will oligomerise into the dimers and tetramers that characterise the common form of AChE in the healthy adult brain. Hence when present within the whole AChE molecule, the C-terminal peptide is directly involved in the oligomerization, so it would *not* be exposed and thus would be unable to bind to the receptor as already identified as responsible for its non-cholinergic actions [26]. It is therefore highly unlikely that the actions of the peptide could occur whilst it is part of the entire protein.

Secondly, what might be the concentration of the peptide in the AD brain, compared to that of AChE itself? If, as work has to date strongly suggested, the C-terminal peptide is indeed a powerful signalling molecule, then it will be removed or broken down very rapidly: hence it would be very hard, indeed impossible, to measure as such in *post mortem* tissue. Nonetheless it is possible that more stable metabolites generated either directly or indirectly by the C-terminal peptide could eventually be identified as a sensitive biomarker ideally detectable in routine blood screens before symptoms present. However such investigations are beyond the scope of the current study, which raises nonetheless the possibility that AChE secretion and the bioactivity of its C-terminal peptides could be additive with Aβ to play a crucial role in the cycle of events characterising neurodegeneration.

## References

[1] Dayan AD (1970) Quantitative histological studies on the aged human brain. I. Senile plaques and neurofibrillary tangles in “normal” patients. Acta Neuropathol 16(2): 85–94. 4919692 10.1007/BF00687663

[2] Greenfield S, Vaux DJ (2002) Parkinson’s disease, AD and motor neurone disease: identifying a common mechanism. Neuroscience 113: 485–492. 12150769 10.1016/s0306-4522(02)00194-x

[3] Farrah T, Deutsch EW, Omenn GS, Campbell DS, Sun Z et al.. (2011) A high-confidence human plasma proteome reference set with estimated concentrations in PeptideAtlas. Mol Cell Proteomics 10: M110 006353-1–M110 006353-14. 10.1074/mcp.M110.006353 PMC3186192 21632744

[4] Greenfield SA, Zimmermann M, Bond CE (2008) Non-hydrolytic functions of acetylcholinesterase. The significance of C-terminal peptides. FEBS J 275: 604–611. 18205834 10.1111/j.1742-4658.2007.06235.x

[5] Greenfield S (1996) Non-classical actions of cholinesterases: role in cellular differentiation, tumorigenesis and AD. Neurochem Int 28: 485–490. 8792328 10.1016/0197-0186(95)00100-x

[6] Bornstein S R, Ehrhart-Bornstein M, Androutsellis-Theotokis A, Eisenhofer G, Vukicevic V, et al (2012) Chromaffin cells: the peripheral brain. Mol Psychiatry 17: 354–358. 22249377 10.1038/mp.2011.176

[7] Averback P (1983) Two new lesions in AD. Lancet 2: 1203. 6139562 10.1016/s0140-6736(83)91256-4

[8] Izumiyama N, Asami E, Itoh Y, Ohtsubo K (1990) Alzheimer’s neurofibrillary tangles and paired helical filaments in the pheochromocytoma cells of the adrenal medulla–electron microscopic and immunoelectron microscopic observations. Acta Neuropathol 81: 213–216. 2127983 10.1007/BF00334510

[9] Takeda M, Tanaka S, Kido H, Daikoku S, Oka M, et al (1994) Chromaffin cells express Alzheimer amyloid precursor protein in the same manner as brain cells. Neurosci Lett 168: 57–60. 8028794 10.1016/0304-3940(94)90415-4

[10] Appleyard ME, McDonald B (1991) Reduced adrenal gland acetylcholinesterase activity in AD. Lancet 338: 1085–1086. 10.1016/0140-6736(91)91947-s 1681391

[11] Atack JR, Perry EK, Perry RH, Wilson ID, Bober MJ, et al (1985) Blood acetyl- and butyrylcholinesterases in senile dementia of Alzheimer type. J Neurol Sci 70: 1–12. 4045496 10.1016/0022-510x(85)90182-0

[12] Berson A, Knobloch M, Hanan M, Diamant S, Sharoni M, et al (2008) Changes in readthrough acetylcholinesterase expression modulate amyloid-beta pathology. Brain 131: 109–119. 18056160 10.1093/brain/awm276

[13] Zimmerman G, Soreq H (2006) Termination and beyond: acetylcholinesterase as a modulator of synaptic transmission. Cell Tissue Res 326: 655–669. 16802134 10.1007/s00441-006-0239-8

[14] Paraoanu LE, Layer PG (2008) Acetylcholinesterase in cell adhesion, neurite growth and network formation. FEBS J 275: 618–624. 18205832 10.1111/j.1742-4658.2007.06237.x

[15] Carvajal FJ, Inestrosa NC (2011) Interactions of AChE with Abeta Aggregates in Alzheimer’s Brain: Therapeutic Relevance of IDN 5706. Front Mol Neurosci 4: 19. 21949501 10.3389/fnmol.2011.00019 PMC3172730

[16] Somogyi P, Chubb IW, Smith AD (1975) A possible structural basis for the extracellular release of acetylcholinesterase. Proc R Soc Lond B Biol Sci 191: 271–283. 2917 10.1098/rspb.1975.0128

[17] Greene LA, Tischler AS (1976) Establishment of a noradrenergic clonal line of rat adrenal pheochromocytoma cells which respond to nerve growth factor. Proc Natl Acad Sci U S A 73: 2424–2428. 1065897 10.1073/pnas.73.7.2424 PMC430592

[18] Mizrachi Y, Naranjo JR, Levi BZ, Pollard HB, Lelkes PI (1990) PC12 cells differentiate into chromaffin cell-like phenotype in coculture with adrenal medullary endothelial cells. Proc Natl Acad Sci U S A 87: 6161–6165. 2117274 10.1073/pnas.87.16.6161 PMC54492

[19] Vichai V, Kirtikara K (2006) Sulforhodamine B colorimetric assay for cytotoxicity screening. Nat Protoc 1(3): 1112–1116. 17406391 10.1038/nprot.2006.179

[20] Decker T, Lohmann-Matthes ML (1988) A quick and simple method for the quantitation of lactate dehydrogenase release in measurements of cellular cytotoxicity and tumor necrosis factor (TNF) activity. J Immunol Methods. 115(1): 61–69. 10.1016/0022-1759(88)90310-9 3192948

[21] Ellman GL (1959) Tissue sulfhydryl groups. Arch Biochem Biophys 82 (1): 70–77. 10.1016/0003-9861(59)90090-6 13650640

[22] Day T, Greenfield SA (2004) Bioactivity of a peptide derived from acetylcholinesterase in hippocampal organotypic cultures. Exp Brain Res 155: 500–508. 14685807 10.1007/s00221-003-1757-1

[23] Onganer PU, Djamgoz MB, Whyte K, Greenfield SA (2006) An acetylcholinesterase-derived peptide inhibits endocytic membrane activity in a human metastatic breast cancer cell line. Biochim Biophys Acta 1760: 415–420. 16469451 10.1016/j.bbagen.2005.12.016

[24] Bond CE, Greenfield SA (2007) Multiple cascade effects of oxidative stress on astroglia. Glia 55: 1348–1361. 17654703 10.1002/glia.20547

[25] Emmett SR, Greenfield SA (2004) A peptide derived from the C-terminal region of acetylcholinesterase modulates extracellular concentrations of acetylcholinesterase in the rat substantia nigra. Neurosci Lett 358: 210–214. 15039118 10.1016/j.neulet.2003.12.078

[26] Greenfield SA, Day T, Mann EO, Bermudez I (2004) A novel peptide modulates alpha7 nicotinic receptor responses: implications for a possible trophic-toxic mechanism within the brain. J Neurochem 90: 325–331. 15228589 10.1111/j.1471-4159.2004.02494.x

[27] Bond CE, Zimmermann M, Greenfield SA (2009) Upregulation of alpha7 Nicotinic Receptors by Acetylcholinesterase C-Terminal Peptides. PLoS One 4, e4846. 10.1371/journal.pone.0004846 PMC2654408 19287501

[28] Zhang XJ, Greenberg DS (2012) Acetylcholinesterase involvement in apoptosis. Front Mol Neurosci 5: 40. 22514517 10.3389/fnmol.2012.00040 PMC3322359

[29] Dinamarca MC, Sagal JP, Quintanilla RA, Godoy JA, Arrázola MS, et al (2010) Mol Neurodegener. 5: 4. 10.1186/1750-1326-5-4 PMC2823746 20205793

[30] Reesa T, Hammonda PI, Soreqc H, Younkinb S, Brimijoina S (2003) Acetylcholinesterase promotes beta-amyloid plaques in cerebral cortex. Neurobiol Aging 24(6): 777–787. 12927760 10.1016/s0197-4580(02)00230-0

[31] Alvarez A, Opazo C, Alarcón R, Garrido J, Inestrosa NC (1997) Acetylcholinesterase promotes the aggregation of amyloid-beta-peptide fragments by forming a complex with the growing fibrils. J Mol Biol 272(3): 348–361. 9325095 10.1006/jmbi.1997.1245

[32] Zbarsky V, Thomas J, Greenfield S (2004) Bioactivity of a peptide derived from acetylcholinesterase: involvement of an ivermectin-sensitive site on the alpha 7 nicotinic receptor. Neurobiol Dis 16(1): 283–289. 15207285 10.1016/j.nbd.2004.02.009

[33] Dineley KT, Bell KA, Bui D, Sweatt JD (2002) Beta -Amyloid peptide activates alpha 7 nicotinic acetylcholine receptors expressed in Xenopus oocytes. J Biol Chem 277 (28): 25056–25061. 10.1074/jbc.M200066200 11983690

[34] Fodero LR, Mok SS, Losic D, Martin LL, Aguilar MI, et al (2004) Alpha7-nicotinic acetylcholine receptors mediate an Abeta(1–42)-induced increase in the level of acetylcholinesterase in primary cortical neurones. J Neurochem 88(5): 1186–1193. 15009674 10.1046/j.1471-4159.2003.02296.x

[35] Nithianantharajah J, Hannan AJ (2011) Mechanisms mediating brain and cognitive reserve: experience-dependent neuroprotection and functional compensation in animal models of neurodegenerative diseases. Prog Neuropsychopharmacol Biol Psychiatry 35: 331–339. 21112312 10.1016/j.pnpbp.2010.10.026

[36] Emery VO (2011) Alzheimer disease: are we intervening too late? Pro. J Neural Transm 118: 1361–1378. 21647682 10.1007/s00702-011-0663-0

[37] Arendt T, Brückner MK, Lange M, Bigl V (1992) Changes in acetylcholinesterase and butyrylcholinesterase in Alzheimer’s disease resemble embryonic development–a study of molecular forms. Neurochem Int 21(3): 381–396. 1303164 10.1016/0197-0186(92)90189-x

